# Effect of Epidermal Growth Factor (EGF) on Cryopreserved Piedmontese Bull Semen Characteristics

**DOI:** 10.3390/ani12223179

**Published:** 2022-11-17

**Authors:** Ahmed R. Alkhawagah, Alessandro Ricci, Penelope Banchi, Nicola A. Martino, Mariagrazia Lucia Poletto, Gian Guido Donato, Tiziana Nervo, Leila Vincenti

**Affiliations:** 1 Theriogenology Department, Faculty of Veterinary Medicine, Benha University, Banha 11629, Egypt; 2 Department of Veterinary Science, University of Torino, Largo Braccini 2, 10095 Grugliasco, Italy; 3 Department of Biosciences, Biotechnologies & Biopharmaceutics, University of Bari Aldo Moro, Via Edoardo Orabona, 70125 Bari, Italy

**Keywords:** bull spermatozoa, cryopreservation, epidermal growth factor, sperm quality, DNA, mitochondrial activity

## Abstract

**Simple Summary:**

This study aimed to investigate bull thawed semen characteristics under the effect of different concentrations (0, 50, 100, 200, and 400 ng/mL) of EGF being added to the extender prior to freezing. Semen samples collected from four bulls for 8 weeks were pooled, diluted with Bullxcell^®^ extender supplemented with EGF at different concentrations, cooled, and frozen. After thawing, semen was examined for different parameters, including motility, kinetics, vitality, integrity, mitochondrial and antioxidant (SOD) activities, mucus penetration distance, as well as in vitro fertilizing capability. EGF incorporation in semen extender improved the total, progressive, and rapid motility and the sperm velocity at 50, 200, and 400 ng/mL after different incubation periods (from 1 to 4 h). We conclude that EGF supplementation to bull sperm extender before cryopreservation has an improving effect on sperm motility and kinetics without affecting sperm parameters.

**Abstract:**

The purpose of this study was to determine the effect on fresh and post-thaw beef bull semen quality of the supplementation of epidermal growth factor (EGF) to the semen extender at various concentrations (0-control, 50, 100, 200, and 400 ng/mL). For 8 weeks, sperm was collected from four fertile bulls, yielding a total of 32 ejaculates. Semen samples were pooled, diluted with Bullxcell^®^ extender, and then cooled, equilibrated, and frozen. After thawing, semen was tested for motility and velocity parameters. Furthermore, semen was evaluated for vitality, integrity, mitochondrial and antioxidant (SOD) activities, mucus penetration distance, and in vitro fertilizing capability. The supplementation with EGF prior to cryopreservation improved the total sperm motility at various concentrations over long incubation periods (from 1 to 4 h). Interestingly, EGF addition improved both progressive and rapid motility, particularly at 50, 200, and 400 ng/mL. In addition, EGF, primarily at 200 and 400 ng/mL, significantly increased several velocity parameters after different incubation periods. We can conclude that adding EGF to bull sperm extender before cryopreservation has a positive stimulatory effect on sperm motility without affecting vitality, integrity, or in vitro fertilizing capability.

## 1. Introduction

Semen cryopreservation is a routine and widespread practice in animal reproduction for its numerous advantages. For instance, it is a key technique for genetic material spread, reducing inbreeding, and improving genetic characteristics within a species or a breed. For this reason, the importance of cryopreserved bull semen is undeniable in the livestock industry [1]. During freezing and thawing procedures, the sperm cells are exposed to biochemical and structural damage to the plasma membrane, acrosome, nucleus, mitochondria, and axoneme. This is caused by temperature and osmotic changes with the formation of ice crystals, increasing oxidative stress and leading to decreased viability, motility, DNA integrity, and reduced sperm fertilizing capability [2,3,4,5,6].

Extenders are liquid diluents intended to protect the spermatozoa during the cryopreservation process [7]. Research on bull semen extenders is considerable, and the effect of implementing various bioactive ingredients to preserve the viability and fertilizing capability of bovine spermatozoa is the main goal [8]. Epidermal growth factor (EGF) is a small protein formed by 53 amino acids (6 kDa), and it can be found in many biosources (e.g., saliva, urines, milk, and blood plasma). Research on EGF in many areas of biology and medicine is increasing, and the involvement of this molecule in mammalian reproduction has been confirmed [9,10]. In males, EGF is produced by Leydig cells and immature germ cells in the testes, and its role in spermatogenesis modulation has been hypothesized [11]. It is involved in the capacitation, acrosomal reaction, and motility characteristics of murine, bovine, and human spermatozoa through the activation of tyrosine kinase [12,13] or protein kinase C [14]. Furthermore, the presence of EGF receptors in the acrosomal region of the spermatozoa has been confirmed in humans, domestic, and laboratory animals [15,16,17]. On ejaculated spermatozoa, the effect of EGF depends on the concentration and time of exposure, other than the animal species [18,19,20]. For instance, the sperm penetrating ability of human spermatozoa is altered when EGF concentration is higher than 25 nM [13], whereas plasma membrane integrity, acrosomal status, and motility of swine spermatozoa are not affected by concentrations as high as 100 ng/mL [17]. In bovines, as in humans, EGF at 10 ng/mL does not affect capacitation nor cause acrosome reaction [13,21]. Nevertheless, lower concentrations (0.1 or 1 ng/mL) were found to induce an acrosomal reaction in bovine spermatozoa [14].

The aim of the present study was to investigate bull thawed semen characteristics when different concentrations (0, 50, 100, 200, and 400 ng/mL) of EGF are added to the extender prior to freezing.

## 2. Materials and Methods

### 2.1. Semen Collection and Extension

Semen samples from four 1.5–2 years old Piedmontese bulls of proven fertility were collected. Included animals were housed at the ANABORAPI Bull Center (Carrù, Piemonte, Italy). Ejaculates were obtained using a bovine artificial vagina, and collection occurred once a week for 8 consecutive weeks (n = 32 ejaculates).

Semen concentration and motility were assessed using a Burker chamber and a Computer Assisted Sperm Analyzer (CASA-Hamilton Thorne, Inc., Beverly, MA, USA) with Leja^®^ slides (Leja, GN Nieuw-Vennep, NL), respectively. Minimum values for inclusion were set at 800 × 10^6^ spz/mL for concentration and 70% for progressive motility.

Suitable samples were pooled and diluted with Bullxcell^®^ extender (IMV, France) to reach a concentration of 30 × 10^6^ spz/mL, following the manufacturer’s instructions. Afterward, extended semen was divided into five experimental aliquots (10 mL each) and supplemented with epidermal growth factor (EGF, E4127, Sigma–Aldrich, Italy) at the following concentrations: 50, 100, 200, and 400 ng/mL; no EGF was added to one aliquot (control). The diluted semen (30 × 10^6^ spermatozoa/mL) was loaded into 0.5 mL plastic straws (Minitube, Germany) for a total of 20 straws per lot and slowly cooled to reach a temperature of 5 °C and equilibrated for 4 hours. Afterward, a programmable machine (SYLAB Gerate GmbH, Neupurkersdorf, Austria) was used to freeze the straws following a standard protocol (Table 1) and then submerge them in liquid nitrogen.

### 2.2. Thawed Semen Evaluation

Straws were thawed in a 37 °C water bath for 30 s, and semen analyses were performed immediately and after 1 h, 2 h, 3 h, and 4 h of incubation in a stove set at 37 °C.

#### 2.2.1. Sperm Concentration and Kinematic

Sperm concentration was manually determined by loading 10 μL of thawed semen into a Burker chamber, and kinetic parameters were assessed by the CASA system, placing 5 μL of semen on a Leja^®^ warmed slide (Leja, GN Nieuw-Vennep, NL), (37 °C). The software setup (Hamilton Thorne, Inc., Beverly, MA, USA) was bull-specific [22].

Eight randomly selected microscopic fields were analyzed, assessing the mean values of the total motility (TM, %), progressive motility (PM, %), rapid motility (RM, %), straight-line velocity (VSL, μm/s), average path velocity (VAP, μm/s), curvilinear velocity (VCL, μm/s), beat cross frequency (BCF, Hz), the amplitude of lateral head displacement (ALH, μm), linearity (LIN, %, [VSL/VCL] × 100), and straightness (STR, %, [VSL/VAP] × 100). The spermatozoa were categorized by the software as fast (>80 μm/s), medium (>60 μm/s), slow (>20 μm/s), or static.

#### 2.2.2. Sperm Viability, Acrosomal Integrity, and Plasma Membrane Integrity

Sperm viability and acrosomal integrity were assessed for all samples using a Trypan Blue/Giemsa staining technique described by Boccia et al. [23]. Briefly, 10 μL of semen and an equal volume of 0.27% Trypan blue stain were mixed and smeared on glass slides. Once dried, the slides were fixed (4 min, 37% formaldehyde with neutral red) and stained overnight with 7.5% Giemsa. A phase contrast microscopy (Advanced Automated Research Microscope System, Nikon Eclipse E200, phase contrast at 40 and 100 magnifications) was used to count 200 sperm cells per slide. Sperm cells were classified based on their staining characteristics: acrosome-intact alive (AIL), acrosome-intact dead (AID), acrosome-lost alive (ALL), and acrosome-lost dead (ALD). Spermatozoa with viable heads and tails were classified as alive, whereas those presenting nonviable heads and/or tails were considered dead.

The plasma membrane integrity of spermatozoa was assessed using a hypo-osmotic swelling assay (HOS) [24]. Specifically, 10 μL of semen were mixed with 100 μL of prewarmed (37 °C) HOS solution (190 mOsm/kg osmotic pressure, containing 1.351 g fructose and 0.735 g sodium citrate dissolved in 100 mL distilled water) and incubated at 37 °C for 40 min. A phase contrast microscope (400×) was used to count 200 spermatozoa after incubation, deeming the percentage of HOS-positive sperm cells with curled tails (swollen/intact plasma membrane).

#### 2.2.3. Flow Cytometric Analysis

Samples were assessed for DNA integrity (sperm chromatin structure assay, SCSA), mitochondrial membrane potential (JC-10 assay), and apoptosis (annexin V/PI binding assay) using a FacsStar Plus^®^ flow cytometer (Becton Dickinson Immunochemistry, San Jose, CA, USA) equipped with standard optics and an air-cooled argon laser operating at 488 nm excitation and 15 mW. A total of 10,000 gated events/s was analyzed at a flow rate of 200 events/set for each sample, and a 488 nm laser was used to excite the sample. CellQuest^®^ software (BD Biosciences, San Jose, CA, USA) was used to collect and analyze the data.

##### DNA Integrity

The SCSA was used to determine the integrity of sperm DNA [25]. Denatured and native DNA was identified by exploiting the metachromatic properties of acridine orange (AO, Sigma-Aldrich, St. Louis, MO, USA). Briefly, the sperm was washed with phosphate-buffered saline (PBS) solution and centrifuged at 1800 RPM for 10 min immediately after thawing. The supernatant was removed, and TNE buffer (0.01 M Tris-Cl, 0.15 M NaCl, 1 mM EDTA, and disodium pH 7.4) was used to dilute sperm pellets to a final concentration of 2 × 10^6^ sperm/mL. Subsequently, 400 µL acid detergent solution (0.08 N HCl, 0.15 M NaCl, 0.1 % (*w/v*) Triton X-100, and pH 1.2) was added, followed by 1200 μL of AO staining solution after 30 s. Flow cytometric evaluations were performed immediately, and red and green fluorescence from the spermatozoa was recorded 3 min after the staining. The fluorescence was measured using FL1 (530/15 nm filter) and FL3 (650 nm long pass filter).

##### Mitochondrial Activity

The lipophilic cation JC-10 was used to measure the mitochondrial activity of spermatozoa (JC-10 Assay for Flow Cytometry, Sigma-Aldrich, St. Louis, MO, USA). JC-10 fluorescence changes reversibly from green (monomeric status) to orange (multimeric status) as mitochondrial membrane potential increases. Briefly, the frozen thawed semen samples were placed into polypropylene tubes at a final concentration of 1 × 10^6^ sperm/mL. After washing with 1 mL PBS and centrifuging for 10 min at 1800 RPM, the pellet was resuspended in 500 µL of JC-10 and incubated for 1 h at 37 °C. Afterward, samples were centrifuged, resuspended in 1 mL of PBS, and analyzed by flow cytometry. Using emission filters of 535 nm and 595 nm, the two populations with green (JC-10 monomers) or orange (JC-10 aggregates) fluorescence were quantified by frequency plots. The percentage of orange-stained sperm was recorded (HMMP), representing a cell population with high mitochondrial membrane potential.

##### Apoptosis (Annexin-V/PI-Binding Assay)

The sperm plasma membrane integrity and translocation of phosphatidylserine (PS) phospholipids were detected using the apoptosis Kit Alexa Fluor 488 Annexin V (V13245, Thermo Fisher Scientific, Waltham, MA, USA) and propidium iodide (PI) [26]. Thawed semen was washed once using PBS and centrifuged at 1800 RPM for 10 min. After the removal of the supernatant, semen pellets were diluted to a final concentration of 1 × 10^6^ spermatozoa/mL in the binding buffer of annexin V (10 mM HEPES, 140 mM NaCl, 2.5 mM CaCl_2_, and pH 7.4). Aliquots of diluted semen (100 μL) were transferred into 5 mL culture tubes and supplemented with 5 μL of annexin V and 1 μL of PI (100 μg/mL). The tubes were gently mixed and incubated at room temperature for 15 min in the dark. Prior to flow cytometric analysis, 400 μL of additional annexin V binding buffer was added to each tube. Forward light scatter (FSC), orthogonal light scatter (SSC), FITC fluorescence (FL1), and PI fluorescence (FL3) were assessed. To exclude debris and other particles from the analysis, an acquisition gate was used in the FSC/SSC two-dimensional histogram. Percentages of viable (annexin V− and PI−), necrotic (annexin V− and PI+), apoptotic (annexin V+ and PI+), and early apoptotic (annexin V+ and PI−) gated spermatozoa were assessed.

#### 2.2.4. Sperm Mucus Penetration Test

The sperm mucus penetration test was performed in a Petri dish, as described by Hossain et al. [27] and Hamano et al. [28]. Briefly, clear cervical mucus was collected from Piedmontese cows in estrus and stored at −80 °C for further use. Sperm Tyrode’s-albumin-lactate-pyruvate (TALP) medium supplemented with 2 mg/mL bovine serum albumin (BSA) was mixed with an equal volume of thawed cervical mucus into a 60 mm Petri dish, forming a horizontal column. Seven 3 cm long, 1–2 mm wide sequentially connected segments formed the column. After coating the column with mineral oil, the Petri dishes were stored in an incubator at 37 °C. Thawed sperm was washed with FERT-TALP by centrifugation at 1500 rpm for 5 min and diluted with BSA containing FERT-TALP to reach a concentration of 40 × 10^6^ spz/mL. Semen (25 µL) was added at the mucous column starting point and incubated at 37 °C for 2 h in a 5 % CO_2_ incubator. Finally, the Petri dish was examined under an inverted microscope (OLYMPUS CK40, Thermo Fisher Scientific, Waltham, MA, USA) to assess sperm migration by measuring the distance between column segments.

#### 2.2.5. Antioxidant Activity (Superoxide Dismutase, SOD)

Semen samples were prepared as described by Sariozkan et al. [29]. Briefly, thawed semen was centrifuged at 800× *g* for 10 min. The sperm pellets were washed 3 times in PBS and resuspended in 1 mL of deionized water. Superoxide dismutase (SOD) activity was assessed using a SOD Assay Kit (19160 SOD determination kit, Sigma–Aldrich, USA). An aliquot (20 μL) of semen was added to 200 μL of reagent, followed by 20 μL of enzyme solution, per the manufacturer’s instructions. After incubation at 37 °C for 20 min, the absorbance was assessed by a microplate reader (INFINITE^®^ F50, TECAN, Mannedorf, Switzerland) at 450 nm, and SOD activity was recorded as u/mL.

### 2.3. Fertilizing Capability

In vitro fertilizing capability was assessed on thawed semen treated with EGF 50 ng/mL and on controls. Briefly, Piedmontese bovine ovaries were collected from the slaughterhouse (Manzo Carni, Cuneo, Italy), washed, and transported in warm saline solution (38 °C) to the lab within 2 h. After rinsing with warm saline, follicles 2–8 mm in diameter were aspirated using an 18 G needle connected to a 10 mL syringe. The follicular fluid sediment was moved into a 100 mm dish, and oocytes with intact compact cumulus were selected and washed three times in TCM-199 HEPES) supplemented with 10% fetal calf serum (FCS) and three times in the in vitro maturation medium. Oocyte maturation occurred in TCM-199 Earle’s salt medium supplemented with 10% FCS, 5 μg/mL luteinizing hormone (LH) (Lutropin-V^®^, Bioniche Animal Health, Belleville, ON, Canada.), 5 μg/mL follicle-stimulating hormone (FSH) (Folltropin-V®, Bioniche Animal Health.), 1 mg/mL 17β-estradiol, 0.2 mM sodium pyruvate, and 10 μg/mL gentamycin [30]. Oocytes were cultured in IVM medium (70 μL droplets, 20 oocytes per droplet), covered with paraffin oil, and maintained at 38.5 °C in 5% CO_2_. After 24 h, thawed semen was washed by centrifugation (30 min at 300 g) using a Percoll discontinuous gradient (45 and 90%). TALP medium supplemented with 1 mM hypotaurine, 250 mM epinephrine, 0.2 mM pyruvate, 2 mM penicillamine, 10 μg/mL gentamicin, 20 μg/mL heparin, and 6 mg/mL BSA was used for in vitro fertilization (IVF). Specifically, semen was added to the droplets containing the oocytes to reach a final concentration of 10 × 106 spermatozoa/mL. After incubation at 38.5 °C for 20 h, cumulus cells were removed, and zygotes were washed and cultured in synthetic oviductal fluid (SOF) [31] at 38.5 °C with 5% CO_2_ and 5% O_2_. After 7 days of incubation, embryos were fixed with 2% paraformaldehyde and stained with Hoechst 33258. Embryos were evaluated for cleavage using a Nikon Eclipse TE 2000-S fluorescence microscope (Nikon^®^, Nikon Solutions Co., Ltd., Shinagawa, Japan) with a B2A (346 nm excitation/460 nm emission) filter.

### 2.4. Statistical Analysis

One-way analyses of variance (ANOVA) with Dunnett’s post hoc test were performed for in-between groups comparisons of motilities (total, progressive, and rapid), velocities (VAP, VS, VCL, ALH, BCF, STR, LIN), sperm viability, the integrity of acrosomes, plasma membrane, and DNA integrity of spermatozoa, sperm apoptosis, mitochondrial membrane potential, mucus penetration distance, and SOD activity. The fertilizing potential was assessed using the Chi-squared test, considering the cleavage rate and blastocyst formation rate. Statistical significance was set for *p* < 0.05. Statistical analyses were performed by SPSS vers. 25 software (IBM^®^, Rome, Italy).

## 3. Results

### 3.1. Motility and Velocity Parameters

Statistically significant differences were found in motility parameters (total, progressive, and rapid) between EGF-extended thawed semen and controls, as reported in Table 2. When differences existed, the semen extended with EGF at different concentrations had higher motility values (Table 2).

Statistically significant differences were found in velocity parameters between EGF-extended thawed semen and controls, as reported in Table 3. When differences existed, the semen extended with EGF at different concentrations had higher velocity values, except for STR and LIN 1h post-thawing when EGF was added to the extender at 50 ng/mL (Table 3).

### 3.2. Sperm Viability and Integrity of Acrosomes, Plasma Membrane, and DNA

Although mean values of viability, acrosome integrity, plasma membrane integrity, and DNA integrity were slightly higher for EGF-extended semen, differences from controls were not significant (*p* > 0.05) (Table 4).

### 3.3. Apoptosis

No differences in the percentage of necrotic and apoptotic cells were found between experimental groups; results are summarized in Table 5.

### 3.4. Sperm Mitochondrial Membrane Potential, Mucus Penetration Distance, and SOD Activity

Although mean values of HMMP, mucus penetration distance, and SOD activity were slightly higher for EGF-extended semen, differences from controls were not significant (*p* > 0.05) (Table 6).

### 3.5. Fertilizing Potential

**Figure 1:**
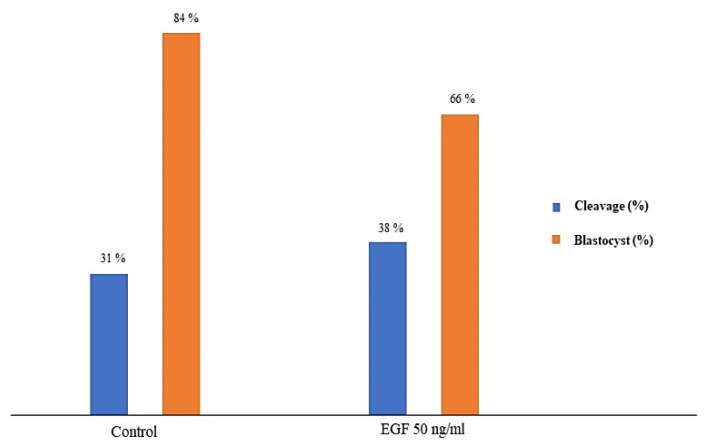
Differences in cleavage and blastocyst formation (%) between in vitro-produced embryos fertilized with thawed semen fertilized in the presence or the absence (controls) of epidermal growth factor (EGF 50 ng/mL). *p* > 0.05.

EGF at a concentration of 50 ng/mL EGF was chosen to test the effect of EGF-treated semen on the fertilizing potential of in vitro-produced embryos. A total of 338 cumulus-oocyte complexes (COCs) were cultured in vitro. Among these, 173 were fertilized with EGF-extended semen, whereas 165 formed the control group. The number of cleaved zygotes was 51 (31%) in the control group and 65 (38%) in the EGF-treated group. The number of blastocysts was 43 (84% from the cleaved zygotes) in the control group and 43 (66 % from the cleaved zygotes) in the EGF-treated group. As shown in Figure 1, no difference was found between the two experimental groups in cleavage and blastocyst formation rates (*p* > 0.05).

## 4. Discussion

The present study aimed to improve the post-thawing fertility potentials of cryopreserved Piedmontese bull semen by supplementing EGF, a cytokine that plays a role in male fertility [10], in the extended semen before cryopreservation. The oxidative stress of sperm membranes caused by the freezing process is known to reduce sperm motility, membrane integrity, and fertilizing ability [32]. Although previous studies reported some positive results on the semen of bulls and males of other species [13,18,33,34], we found none-to-modest differences in sperm viability, membrane and DNA integrity, and fertilizing potential compared with thawed semen that had not been extended with EGF. Nevertheless, the results of the present research revealed that EGF at different concentrations has a mild positive effect on sperm motility and velocity parameters immediately after thawing and after different times of incubation. Specifically, the best effects were recorded with the concentration of EGF equal to 50, 200, and 400 ng/mL. This result is consistent with previous literature reporting that EGF at concentrations of 200 and 400 ng/mL significantly improved the total and progressive motilities of cooled ram sperm [32]. In boars, EGF concentrations equal to 100 ng/mL improved the quality of sperm movement [17], whereas, in buffalo, a numerical increase in sperm motility at concentrations equal to 50, 100, and 200 ng/mL was described [33]. Furthermore, Naz and Kaplan [13] reported that concentrations of EGF as low as 25–50 nM improved human sperm velocity and ALH, whereas concentration equal to 100 nM improved all kinematic parameters. The exact mechanism by which EGF influences sperm motility has not been clarified. This effect may be mediated via Ras or Rho components of the ERK pathway, which are localized in the flagellum of spermatozoa in hamsters [35] and bulls [36] and are involved in sperm motility regulation. The gradual decrease in kinematic parameters at different incubation periods may be associated with mitochondrial aging that leads to a decreased ability of the spermatozoa to produce the adenosine triphosphate (ATP) [37].

It has been reported that sperm with higher velocities have higher fertilization potential in bovines [38]. However, sperm motility evaluation is not sufficient for sperm selection, as low motility samples could be balanced by a low content in apoptotic and/or necrotic spermatozoa, resulting in a good potential for insemination [39]. Therefore, sperm motility evaluation should be combined with the determination of apoptotic or necrotic changes [32]. In the present study, no difference was found in the percentage of necrotic and apoptotic sperm cells in-between experimental groups. However, a numerical decrease in the percentage of apoptotic spermatozoa was recorded in the EGF-treated groups compared with the control. This is coherent with results by Makarevich et al. [32] for ram semen.

Mitochondrial status plays an important role in sperm fertility because of its relationship with the energetic status and motility of the spermatozoon [40,41]. Sperm mitochondrial transmembrane potential is considered one of the most sensitive tests for semen quality assessment and qualification for field fertility [42]. It has been recorded that the sperm with HMMP positively correlated with in vivo fertility in buffalo during the low breeding season [41,43] and with high motility values of frozen–thawed ram semen [44]. In the present study, the proportion of sperm with HMMP numerically increased in all groups upon EGF treatments.

For pregnancy achievement, spermatozoa must travel a considerable distance to meet the ovum at the natural fertilization site, the fallopian tube [16]. Some studies indicate that sperm motility is affected by the muscular contraction of the reproductive tissue and by the stimulatory/inhibitory influences of its fluid environment [17]. The Petri dish-based horizontal column containing cervical mucus may be a reliable tool for effectively analyzing sperm migration, and it has the potential as a predictor of sperm fertility [26,27]. In fact, a positive correlation has been found between bull sperm viability after migration in cervical mucus-supplemented media and fertility [27]. In the present study, we recorded a numerical increase in the mucus penetration distance in all EGF-treated samples.

As mentioned, sperm freezing–thawing procedures have been associated with excessive production of reactive oxygen species (ROS) [17]. It has been reported that EGF contains six half-cysteine residues [45], which act as nonenzymatic antioxidants, and easily penetrate the sperm cell [46]. Our results revealed a numerical increase in the SOD activity in the 100 and 200 ng/mL EGF groups compared with the others. This is coherent with previous research [32] that investigated frozen–thawed buffalo sperm, finding an increase in SOD activity by adding EGF at 100 ng/mL and 200 ng/mL concentrations. As hinted in the present study, EGF addition to the extender failed to improve the in vitro fertilizing capability of thawed bull sperm. To the authors’ knowledge, there is no previous report describing the effect of EGF treatment on sperm fertilizing capacity. Nonetheless, the literature showed that EGF addition improved the in vitro maturation, cleavage, and blastocyst rates when used during in vitro maturation in bovine oocytes [47,48].

In conclusion, semen extended with EGF at concentrations equal to 50, 200, and 400 ng/mL before cryopreservation resulted in a modest improvement in different sperm motility and velocity parameters. Furthermore, it led to an improvement in vitality, HMMP, SOD activity, and mucus penetration distance of the sperm, also decreasing the percentage of apoptotic sperm cells without affecting sperm integrity and in vitro fertilizing capability. Further studies are needed to determine the effect of EGF in bulls with reduced fertility.

## Figures and Tables

**Table 1:** The freezing protocol set on a programmable freezing machine (SYLAB Gerate GmbH, Neupurkersdorf, Austria).

Temperature Interval	Rate
+4 °C to −9 °C	−4 °C/min
−9 °C to −25 °C	−50 °C/min
−25 °C to −100 °C	−35 °C/min
−100 °C to −144 °C	−20 °C/min
−144 °C to −150 °C	−4 °C/min

**Table 2:** Differences in motility parameters (total, progressive, and rapid) between thawed semen extended with epidermal growth factor (EGF) at different concentrations (50, 100, 200, and 400 ng/mL) and controls (without EGF) at different post-thaw times (n = 8 replicates).

Parameter	Group	Post-Thaw (Mean ± SEM)	1 h (Mean ± SEM)	2 h (Mean ± SEM)	3 h (Mean ± SEM)	4 h (Mean ± SEM)
Total motility (%)	control	90.36 ± 0.58	83.38 ± 0.87 ^a^	67.78 ± 1.67	50.31 ± 1.41 ^a^	43.05 ± 1.21 ^a^
	EGF 50 ng/mL	89.71 ± 1.00	89.97 ± 0.83 ^b^	69.00 ± 1.38	53.91 ± 1.10	44.91 ± 1.14
	EGF 100 ng/mL	91.00 ± 0.33	85.53 ± 0.69	67.97 ± 1.19	56.39 ± 1.17 ^b^	46.42 ± 1.41
	EGF 200 ng/mL	91.98 ± 0.51	85.61 ± 0.04	70.17 ± 1.34	52.67 ± 1.19	48.58 ± 1.08 ^b^
	EGF 400 ng/mL	88.88 ± 1.18	86.34 ± 0.56 ^b^	71.23 ± 1.36	52.44 ± 0.85	45.09 ± 1.55
	Significance	NS	0.001	NS	0.001	0.01
Progressive motility (%)	control	53.19 ± 0.66	43.56 ± 0.70 ^a^	26.63 ± 1.30 ^a^	6.16 ± 1.40	4.02 ± 1.25
	EGF 50 ng/mL	53.03 ± 0.85	44.56 ± 1.00	30.78 ± 1.14 ^b^	9.28 ± 1.26	3.84 ± 0.99
	EGF 100 ng/mL	54.12 ± 0.57	45.58 ± 0.64	27.20 ± 0.98	8.38 ± 1.29	4.09 ± 1.16
	EGF 200 ng/mL	54.69 ± 0.58	48.09 ± 0.71 ^b^	32.77 ± 1.01 ^b^	9.89 ± 1.45	3.66 ± 0.96
	EGF 400 ng/mL	54.44 ± 0.97	44.89 ± 0.60	32.47 ± 1.39 ^b^	8.06 ± 1.00	4.75 ± 1.34
	Significance	NS	0.001	0.001	NS	NS
Rapid motility (%)	control	72.09 ± 0.54	61.94 ± 0.88 ^a^	41.77 ± 1.56 ^a^	18.84 ± 2.20	10.34 ± 2.10
	EGF 50 ng/mL	71.56 ± 1.00	66.77 ± 1.33 ^b^	46.72 ± 1.16 ^b^	23.91 ± 0.88	11.83 ± 1.59
	EGF 100 ng/mL	71.25 ± 0.60	64.53 ± 0.83	44.33 ± 1.15	24.95 ± 1.98	11.89 ± 2.25
	EGF 200 ng/mL	73.23 ± 0.60	67.02 ± 1.06 ^b^	48.20 ± 0.99 ^b^	25.08 ± 1.98	14.08 ± 1.98
	EGF 400 ng/mL	71.44 ± 1.25	63.31 ± 0.69	49.31 ± 1.69 ^b^	24.53 ± 1.56	13.41 ± 2.10
	Significance	NS	0.001	0.001	NS	NS

NS—Not significant. The superscripts within the same column indicate significant differences versus controls.

**Table 3:** Differences in velocity parameters between thawed semen extended with epidermal growth factor (EGF) at different concentrations (50, 100, 200, and 400 ng/mL) and controls (without EGF) at different post-thaw times (n = 8 replicates).

	Group	Post-Thawing (Mean ± SEM)	1 h (Mean ± SEM)	2 h (Mean ± SEM)	3 h (Mean ± SEM)	4 h (Mean ± SEM)
VAP (μm/s)	control	87.01 ± 0.38 ^a^	80.01 ± 0.433 ^a^	60.52 ± 1.06 ^a^	50.55 ± 1.22 ^a^	49.33 ± 1.33
	EGF 50 ng/mL	86.49 ± 0.42	80.65 ± 0.42	63.13 ± 0.78	54.10 ± 1.31	49.18 ± 1.19
	EGF 100 ng/mL	87.32 ± 0.28	80.81 ± 0.38	63.38 ± 1.09	53.16 ± 0.12	47.99 ± 1.27
	EGF 200 ng/mL	87.69 ± 0.21	81.78 ± 0.42 ^b^	64.56 ± 0.92 ^b^	54.35 ± 0.15	49.67 ± 1.05
	EGF 400 ng/mL	89.56 ± 0.22 ^b^	82.34 ± 0.39 ^b^	63.64 ± 1.13	55.33 ± 1.14 ^b^	50.14 ± 1.35
	Significance	0.001	0.001	0.01	0.01	NS
VSL (μm/s)	control	72.26 ± 0.24 ^a^	65.65 ± 0.43 ^a^	49.10 ± 0.85 ^a^	35.50 ± 1.201 ^a^	32.00 ± 1.35
	EGF 50 ng/mL	71.99 ± 0.24	65.02 ± 0.34	51.34 ± 0.69	39.72 ± 1.32 ^b^	32.09 ± 1.31
	EGF 100 ng/mL	73.48 ± 0.16 ^b^	66.22 ± 0.22	51.16 ± 0.85	37.87 ± 1.18	31.71 ± 1.19
	EGF 200 ng/mL	73.32 ± 0.20 ^b^	67.31 ± 0.36 ^b^	52.80 ± 0.77 ^b^	39.58 ± 1.19	32.60 ± 1.10
	EGF 400 ng/mL	75.51 ± 0.27 ^b^	67.45 ± 0.23 ^b^	52.08 ± 0.87 ^b^	39.81 ± 1.28 ^b^	32.73 ± 1.44
	Significance	0.001	0.001	0.01	0.05	NS
VCL (μm/s)	control	143.20 ± 1.07	137.54 ± 0.77 ^a^	102.98 ± 1.65 ^a^	89.11 ± 2.05 ^a^	81.28 ± 2.41
	EGF 50 ng/mL	141.92 ± 1.20	137.86 ± 0.88	107.82 ± 1.35	95.52 ± 2.09	80.91 ± 2.22
	EGF 100 ng/mL	141.82 ± 0.85	139.58 ± 0.89	108.64 ± 1.82	93.24 ± 1.84	80.47 ± 2.44
	EGF 200 ng/mL	143.76 ± 0.68	141.47 ± 0.94 ^b^	110.18 ± 1.71 ^b^	96.63 ± 1.66 ^b^	84.40 ± 2.02
	EGF 400 ng/mL	144.73 ± 0.62	141.71 ± 0.81 ^b^	108.52 ± 1.96	98.24 ± 1.97 ^b^	85.01 ± 2.55
	Significance	NS	0.001	0.01	0.01	NS
ALH (μm)	control	5.98 ± 0.05	6.10 ± 0.02 ^a^	5.54 ± 0.06	5.09 ± 0.35	2.40 ± 0.28 ^a^
	EGF 50 ng/mL	5.90 ± 0.06	6.14 ± 0.03	5.70 ± 0.08	5.47 ± 0.27	4.29 ± 0.34 ^b^
	EGF 100 ng/mL	5.84 ± 0.05	6.13 ± 0.03	5.64 ± 0.05	5.61 ± 0.30	3.83 ± 0.41 ^b^
	EGF 200 ng/mL	5.96 ± 0.03	6.19 ± 0.03	5.57 ± 0.45	5.47 ± 0.22	4.31 ± 0.43 ^b^
	EGF 400 ng/mL	5.93 ± 0.03	6.23 ± 0.04 ^b^	5.37 ± 0.05	5.54 ± 0.25	4.28 ± 0.38 ^b^
	Significance	NS	0.01	NS	NS	0.001
BCF (Hz)	control	27.95 ± 0.22	22.31 ± 0.13 ^a^	18.50 ± 0.21 ^a^	14.12 ± 0.34 ^a^	14.03 ± 0.33
	EGF 50 ng/mL	27.48 ± 0.18	22.26 ± 0.13	19.07 ± 0.23	14.95 ± 0.37	14.80 ± 0.43
	EGF 100 ng/mL	28.04 ± 0.23	22.90 ± 0.13 ^b^	18.89 ± 0.20	15.14 ± 0.40	13.12 ± 0.35
	EGF 200 ng/mL	28.14 ± 0.14	22.72 ± 0.16	19.63 ± 0.17 ^b^	15.61 ± 0.42 ^b^	13.97 ± 0.37
	EGF 400 ng/mL	28.56 ± 0.23	23.01 ± 0.15 ^b^	19.73 ± 0.26 ^b^	15.45 ± 0.34 ^b^	14.75 ± 0.32
	Significance	NS	0.01	0.001	0.05	NS
STR (%)	control	83.42 ± 0.21 ^a^	82.30 ± 0.41 ^a^	82.13 ± 0.35	70.64 ± 0.62 ^a^	64.75 ± 0.74
	EGF 50 ng/mL	83.69 ± 0.28	80.88 ± 0.17 ^b^	82.02 ± 0.28	73.52 ± 0.73 ^b^	64.92 ± 0.84
	EGF 100 ng/mL	84.38 ± 0.21 ^b^	82.53 ± 0.19	81.64 ± 0.21	71.75 ± 0.82	66.48 ± 0.62
	EGF 200 ng/mL	83.98 ± 0.28	82.72 ± 0.14	82.48 ± 0.21	73.25 ± 0.76 ^b^	65.70 ± 0.74
	EGF 400 ng/mL	84.56 ± 0.38 ^b^	82.11 ± 0.19	82.77 ± 0.27	72.03 ± −0.91	64.91 ± 0.85
	Significance	0.05	0.001	NS	0.05	NS
LIN (%)	control	52.59 ± 0.29 ^a^	48.95 ± 0.24 ^a^	49.14 ± 0.30	40.33 ± 0.44	40.03 ± 0.45
	EGF 50 ng/mL	52.80 ± 0.38	48.33 ± 0.15 ^b^	49.11 ± 0.32	42.06 ± 0.55	39.73 ± 0.46
	EGF 100 ng/mL	53.63 ± 0.31	48.94 ± 0.19	48.53 ± 0.21	41.30 ± 0.57	40.19 ± 0.42
	EGF 200 ng/mL	53.09 ± 0.32	48.94 ± 0.16	49.45 ± 0.22	41.70 ± 0.63	39.25 ± 0.40
	EGF 400 ng/mL	54.05 ± 0.34 ^b^	48.94 ± 0.16	49.55 ± 0.30	41.08 ± 0.60	38.86 ± 0.47
	Significance	0.01	0.05	NS	NS	NS

NS: Not significant. VAP: Average path velocity (μm/s). VSL: Straight linear velocity (μm/s). VCL: Curvilinear velocity (μm/s). ALH: Amplitude of lateral head displacement (μm). BCF: Beat cross frequency (Hz). STR: straightness ([VSL/VAP] × 100). LIN: Linearity ([VSL/VCL] × 100). The superscripts within the same column indicate statistically significant differences versus controls.

**Table 4:** Differences in vitality, acrosome, DNA integrity, and plasma integrity between thawed semen extended with epidermal growth factor (EGF) at different concentrations (50, 100, 200, and 400 ng/mL) and controls (without EGF) at different post-thaw times.

Group	Sperm Vitality (%) (Mean ± SEM)	Acrosome Integrity (%) (Mean ± SEM)	Plasma Membrane Integrity (%) (Mean ± SEM)	DNA Integrity (%) (Mean ± SEM)
Control	68.56 ± 1.32	90.94 ± 1.75	60.94 ± 1.65	94.02 ± 0.35
EGF 50 ng/mL	65.50 ± 1.10	90.56 ± 1.71	61.94 ± 1.95	93.85 ± 0.70
EGF 100 ng/mL	70.06 ± 1.34	92.25 ± 1.52	64.63 ± 1.74	94.02 ± 0.66
EGF 200 ng/mL	72.38 ± 1.50	92.06 ± 1.78	63.00 ± 2.35	94.25 ± 0.63
EGF 400 ng/mL	71.06 ± 1.50	93.56 ± 1.36	62.88 ± 1.95	93.43 ± 0.73
Significance	NS	NS	NS	NS

NS: Not significant.

**Table 5:** Differences in apoptosis between thawed semen extended with epidermal growth factor (EGF) at different concentrations (50, 100, 200, and 400 ng/mL) and controls (without EGF) at different post-thaw times (n = 8 replicates).

Group	Viable Sperm (%) (Mean ± SEM)	Necrotic Sperm (%) (Mean ± SEM)	Apoptotic Sperm (%) (Mean ± SEM)
Control	45.95 ± 10.65	45.90 ± 5.61	8.15 ± 5.41
EGF 50 ng/mL	50.41 ± 5.43	47.78 ± 5.28	1.80 ± 0.34
EGF 100 ng/mL	52.69 ± 3.64	46.29 ± 3.70	1.02 ± 0.23
EGF 200 ng/mL	51.63 ± 3.64	47.36 ± 3.88	1.02 ± 0.31
EGF 400 ng/mL	50.54 ± 2.85	48.48 ± 2.81	1.15 ± 0.23
Significance	NS	NS	NS

NS: Not significant.

**Table 6:** Differences in sperm mitochondrial membrane potential, mucus penetration distance, and SOD activity between thawed semen extended with epidermal growth factor (EGF) at different concentrations (50, 100, 200, and 400 ng/mL) and controls (without EGF) at different post-thaw times (n = 8 replicates).

Group	HMMP (%) (Mean ± SEM)	Mucus Penetration Distance (cm) (Mean ± SEM)	SOD Activity (u/mL) (Mean ± SEM)
Control	12.44 ± 1.68	8.75 ± 1.93	1.68 ± 0.02
EGF 50 ng/mL	24.15 ± 12.44	10.00 ± 1.78	1.67 ± 0.04
EGF 100 ng/mL	30.67 ± 9.39	9.73 ± 1.98	1.72 ± 0.03
EGF 200 ng/mL	25.73 ± 10.35	10.38 ± 1.14	1.69 ± 0.03
EGF 400 ng/mL	22.61 ± 10.30	9.70 ± 0.83	1.64 ± 0.04
Significance	NS	NS	NS

NS: Not significant.

## Data Availability

Not applicable.
